# Greenstick fracture of the ulnar shaft following physical therapy in an adult: A case report

**DOI:** 10.1097/MD.0000000000023612

**Published:** 2020-12-11

**Authors:** Yi-Chen Lin, Wei-Te Wang

**Affiliations:** Department of Physical Medicine and Rehabilitation, Changhua Christian Hospital, Changhua, Taiwan.

**Keywords:** greenstick fractures, physical therapy, post-internal fixation

## Abstract

**Rationale:**

Greenstick fractures most commonly occur in the pediatric population, especially in those under 10 years of age. Greenstick fractures are “extremely” rare in adults. This report presents the case of a greenstick fracture of the ulnar shaft in an adult following physical therapy for a radial neck fracture and ulnar shaft fracture post-internal fixation. Greenstick fracture can occur during physical therapy near the drill holes created during surgery.

**Patient concerns:**

A 23-year-old man without any past medical history had sustained a greenstick fracture of the ulnar shaft after rehabilitation for a left radial and ulnar fracture that had been previously treated with internal fixation.

**Diagnoses:**

Five months after removal of the implants, the patient complained of left elbow tenderness and a “breaking” sound that occurred during physical therapy. The results of a subsequent X-ray revealed a greenstick fracture of the left ulnar shaft.

**Interventions:**

Splinting of the fracture.

**Outcomes:**

After 2 months of splint fixation, the pain and range of motion in the affected arm were improved, and sequential X-rays showed callus formation and increased density of the ulnar shaft.

**Lessons:**

Greenstick fractures occur not only in children but also in adults in specific circumstances. The cortex of long bones may be further weakened by drill holes created during surgery, and fractures may occur during physical therapy. During treatment, physicians, and therapists should pay more attention to the patient who has undergone implant removal to avoid greenstick fractures, especially in the locations near drill holes.

## 1 Introduction

Greenstick fractures occur in around 1 in 100 children every year, and are most common in children aged between 5 and 14 years.^[1]^ However, they are also seen in other age groups due to sports injuries or traffic accidents, for example. Among the different fracture patterns of children, greenstick fractures comprise only 5.27% and are extremely rare in adults.^[2]^ This report presents a case of a greenstick fracture of the ulnar shaft in an adult that followed physical therapy for left radius and ulnar fractures post-internal fixation. We subsequently discuss the possible causes of the fracture, which may have been attributed to the drill holes or manipulation of physical therapy. The fracture was treated with splinting and sequential X-rays, which showed callus formation and increased density of the ulnar shaft. This report conforms to all CARE guidelines and reports the needed information accordingly. Written informed consent was obtained from the patient for publication of this report.

## 2 Method

This is a report of a case under the waiver of approval of the ethics committee or institutional review board. Written informed consent was obtained from the patient for the publication of this report.

## 3 Case presentation

**Figure 1:**
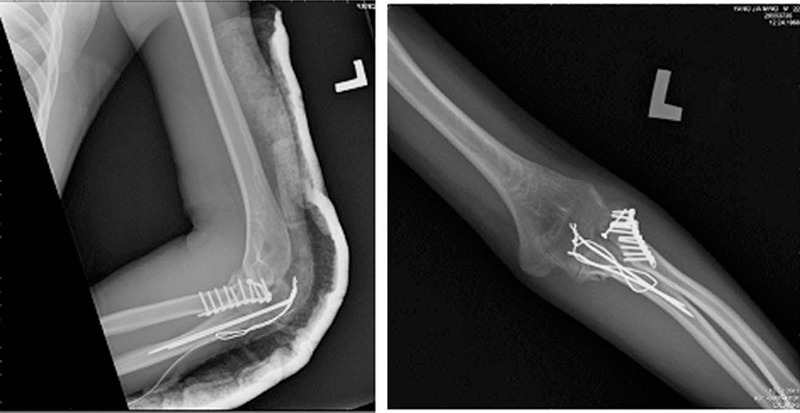
Underwent open reduction and internal fixation for a radial neck fracture and left olecranon fracture.

A 23-year-old man, without any past medical history, underwent open reduction, and internal fixation for a radial neck fracture and left olecranon fracture (Fig. 1) sustained during a fall. He was referred to the rehabilitation department due to pain and a limited range of motion (ROM) of his left elbow 2 months following the injury. Physical examination of the left elbow in the clinic of the rehabilitation department revealed an active ROM of 40° to 60°, passive ROM of 40° to 60°, supination of 0° to 20°, and pronation of 0° to 30°. Physical therapy was arranged with therapeutic exercises including joint mobilization, stretch exercises to help increase elbow extension, as well as elbow flexion and extension with a sand bag and wrist supination and pronation with a stick following 15 minutes of infrared therapy.

**Figure 2:**
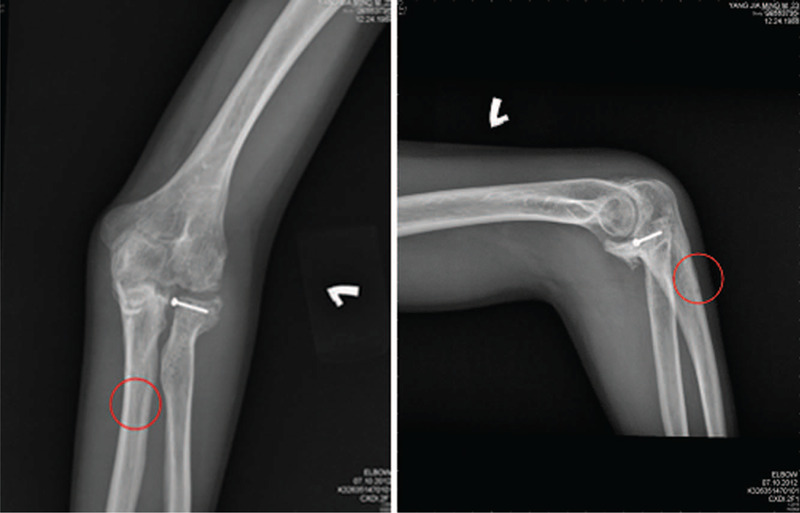
The post removal of the internal fixation X-ray showed the bone union of the fracture site and smooth cortical bone without obvious fracture around the previous fracture site or the drill holes. The drill hole (the red circle) is visible after removal of the screw.

After approximately 6 months of physical therapy, the pain and ROM in the elbow improved, with an active ROM of 40° to 100°, passive ROM of 5° to 110°, supination of 0° to 30°, and pronation of 0° to 35°. He was admitted again 9 months after surgery to undergo removal of the internal fixation, and was discharged home after 2 days. During admission for removal, an X-ray taken showed bony union of the fracture site and smooth cortical bone without obvious fractures around the previous injury site or the drill holes (Fig. 2). After discharge, the patient remained under the rehabilitation program at our department and the symptoms gradually improved.

**Figure 3:**
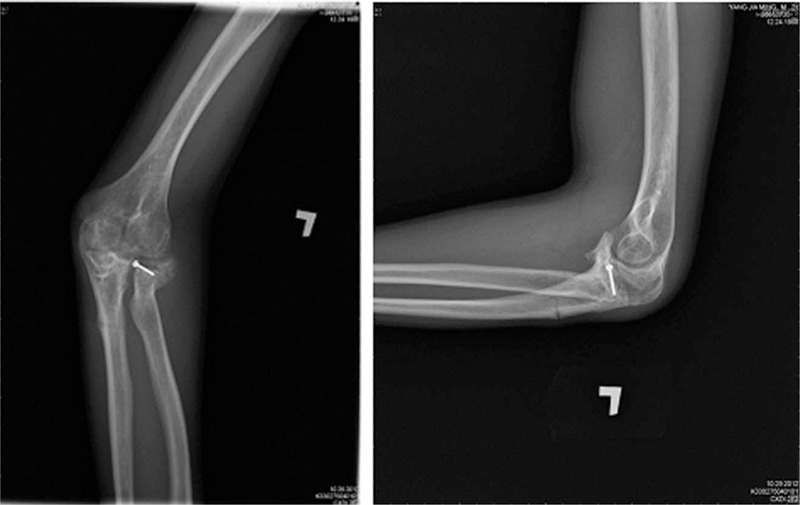
Five months after the removal of the internal fixation and after “breaking” sound, subsequent X-ray revealed a greenstick fracture of the left ulnar shaft.

**Figure 4:**
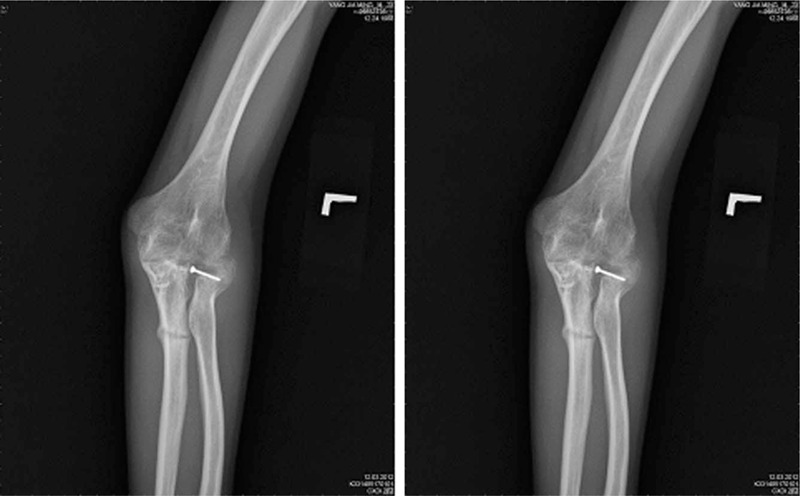
Callus and increased density of ulnar shaft was identified on the subsequent X-rays.

However, on 1 day, 5 months after removal of the internal fixation, the patient complained of left elbow tenderness with a “breaking” sound occurring during physical therapy. He was promptly sent to emergency room in our hospital and the results of a subsequent X-ray revealed a greenstick fracture of the left ulnar shaft (Fig. 3). After discussion with our orthopedic department, a cross-elbow splint was applied. Two months after splinting, the tenderness and numbness of the elbow was alleviated, and callus and increased density of the ulnar shaft was identified on subsequent X-rays (Fig. 4).

## 4 Discussion and conclusion

There are differences between adult and child bone, due to the presence of the epiphyseal plate in the pediatric population. The epiphyseal plate allows bone growth from a cartilage base, and after endochondral ossification the cartilage is replaced by primary bone.^[3]^ There are 2 reasons why greenstick fractures are more prevalent in children. First, the pediatric bone consists of calcified cartilage, which is flexible compared to the ossified adult bone, and pediatric bone tends to be more susceptible to bowing injuries under tension. Second, the periosteum is more tough and thicker in children.^[4]^

**Figure 5:**
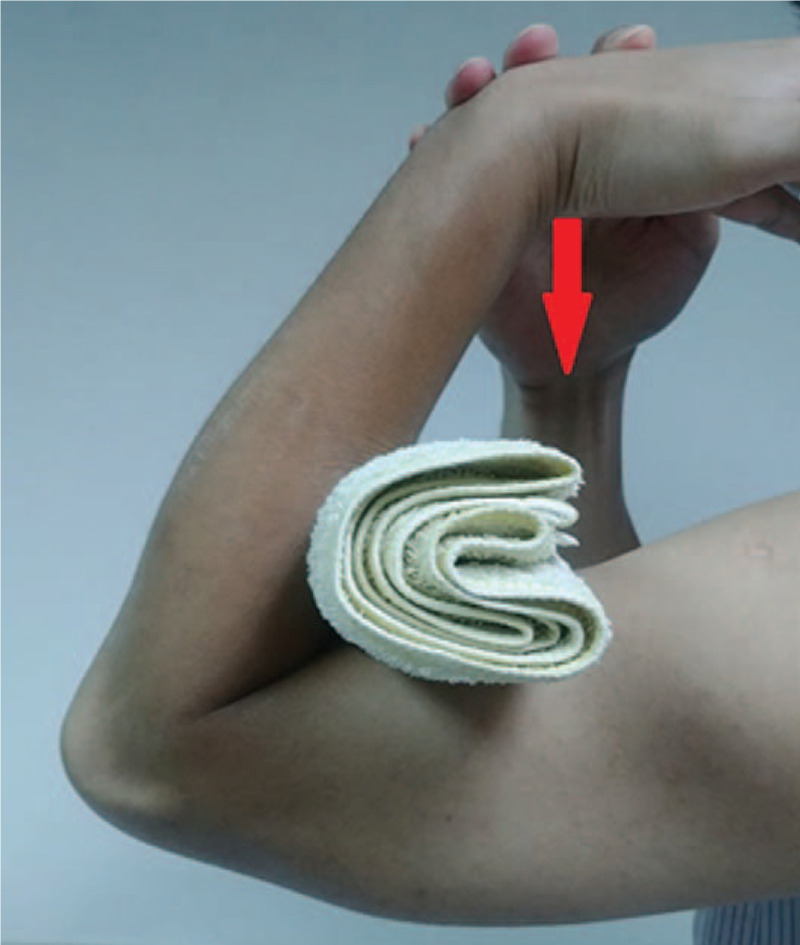
Joint mobilization. The force (the red arrow) applied from the physical therapist during treatment.

The mechanism underlying the development of a greenstick fracture in our adult patient could be 2-fold. First, the cortex of the long bone may have been weakened by the drill holes from surgery. After removal of the screw, the holes may have been initially filled with dense woven bone, which was slowly remodeled into cortical bone. Several studies have confirmed the strength of long bones decreases after drill hole placement.^[5–7]^ The drilled bone can only absorb a low amount of energy prior to failing.^[5]^ Band wires may further weaken the drilled bone, and radiographs are not sensitive for the detection of narrow caliber bone.^[8]^ The drill hole was visible after removal of the screw (Fig. 2; red circle). The second potential underlying mechanism for the development of a greenstick fracture in this case was that during joint mobilization (Fig. 5), large torque may have been induced over the bone. The force applied from physical therapy (Fig. 5; red arrow) may have caused the ulnar bone to act like a lever. When the pivot was located in the defect induced by the screw, the fracture may have resulted.

Greenstick fractures can be treated with splinting, if the patient and/or family members monitor the fracture closely.^[9]^ Acceptable alignment of the fragments can result from this strategy.^[10]^ Immobilization of greenstick fractures should occur for approximately 6 weeks.^[11]^ In our case, after 2 months of splint fixation, the pain and range of motion were improved, and sequential X-rays showed callus formation and increased density of the ulnar shaft. Greenstick fractures not only occur in children but also in adults. The cortex of long bones may be further weakened by drill holes performed during surgery, and fractures may occur during joint mobilization, when the pivot is located in a weak point of the bone. This highlights the importance of extra care during treatment. During treatment, physicians, and therapists should pay greater attention to a patient undergoing internal fixation to avoid greenstick fracture, especially in the location around drill holes. In addition, splinting appears to be a feasible option for the management of greenstick fractures.

## Author contributions

**Conceptualization:** Yi-Chen Lin, Wei-Te Wang.

**Supervision:** Wei-Te Wang.

**Writing – original draft:** Yi-Chen Lin

**Writing – review & editing:** Wei-Te Wang.
